# Urine biomarkers for gentamicin-induced acute kidney injury in the neonatal ICU

**DOI:** 10.1186/cc9526

**Published:** 2011-03-11

**Authors:** D Jansen, S Heemskerk, L Koster-Kamphuis, TP Bouw, AF Heijst, P Pickkers

**Affiliations:** 1 Radboud University Nijmegen Medical Centre, Nijmegen, the Netherlands

## Introduction

Gentamicin (GM) is an aminoglycoside frequently used in the neonatal ICU to treat infections. Despite low resistance and costs, GM is also nephrotoxic and may cause acute kidney injury (AKI). Serum creatinine appears to be an insensitive and unreliable marker in this setting. The objective of this study was to determine whether urine biomarkers are useful for early detection of gentamicin-induced AKI in neonates in the neonatal ICU.

## Methods

### Subjects

Thirty-three neonates (26 male/seven female, gestational age ± 36 weeks) with a bladder catheter without pre-existent kidney disease were divided into a GM group (*n *= 20) and a reference group (*n *= 13).

### Study design and procedures

A prospective, clinical observational trial with non-invasive procedures. Demographics, vital signs and clinical conditions were recorded. Every 2 hours, during the period of bladder catheter, urine samples were collected and renal injury biomarkers glutathione-*S*-transferase A1-1 (GSTA1-1), GSTP1-1, kidney injury marker-1 (KIM-1), *N-*acetyl-β-D-glucosaminidase (NAG) and neutrophil gelatinase-associated lipocalin (NGAL) were determined. Residual blood samples were used to measure serum creatinine (sCr).

## Results

Demographics were similar between both groups expect for baseline BUN (*P *< 0.04), which disappeared after the first day of the study. No significant differences were found in baseline kidney function, hemodynamics, ventilation support and reason for admission. Treatment with GM resulted in higher levels of sCr compared with the reference group (58.5 (44.8 to 78.5) vs. 34 (28.3 to 58.8) mmol/l; *P *< 0.05). The average time until the highest peak was shorter for both GSTA1-1 and GSTP1-1 compared with sCr (*P *< 0.05). Furthermore, higher levels of sCr corresponded with higher levels of urine biomarkers and KIM-1, NAG and GSTP1-1 could differentiate between the GM and reference group.

## Conclusions

Higher sCr levels correspond with higher urinary excretion of all biomarkers, especially after GM use. In addition, the urinary biomarker GSTP1-1 might be useful for early detection of AKI in the neonatal ICU.

